# Critical factors in the successful expansion of Pneumococcal Conjugate Vaccine in India during the COVID-19 pandemic

**DOI:** 10.1016/j.jvacx.2023.100328

**Published:** 2023-06-05

**Authors:** Seema Singh Koshal, Arindam Ray, Rhythm Hora, Amanjot Kaur, Syed F Quadri, Rashmi Mehra, Amrita Kumari, Pradeep Haldar, Arup Deb Roy

**Affiliations:** a John Snow India, India; b Bill and Melinda Gates Foundation, India; c Former Advisor (RCH), Ministry of Health & Family Welfare, India

**Keywords:** COVID-19, Pneumococcal Conjugate Vaccine (PCV), World Health Organization (WHO)

## Abstract

India had decided to roll out PCV in India in 2015, but successful implementation of any new vaccine introduction mandates an enormous effort. PCV was scaled up during the COVID-19 pandemic, which posed new, unprecedented challenges in the vaccine rollout. However, India successfully expanded PCV in the country in record time across all states and Union Territories.

During the pandemic, supply-side restrictions, delayed vaccine shipments, staff shortages, and restrictions in conducting training negatively affected the roll-out of PCV across the country. However, despite the ongoing pandemic, India successfully rolled out PCV across the country in 7 months. In this review, the authors have conducted a narrative review to delineate the crucial factors that helped in the successful expansion of PCV.

## Highlights

• The nationwide rollout of the Pneumococcal Conjugate Vaccine (PCV) during the pandemic demonstrates the resilience of the health system.
• The study highlights five critical factors that have been the key to the rollout of PCV in India.
• The study draws attention to the fact that India has nurtured a robust health system that is all set to tackle adverse situations.

## Introduction

COVID-19 has affected all countries in the world; however, it presented the biggest threat to low and middle-income countries battling the pandemic and trying to enhance the capacity of health systems and innovative strategies for achieving a functional health system along with COVID-19 services [1].

A study estimated that in 2010, pneumococcus was responsible for at least 0.56 million severe episodes of pneumococcal pneumonia and 0.1 million pneumococcal deaths occurred in India in children under the age of 5 years [2]. In 2015, the National Technical Advisory Group on Immunization (NTAGI) recommended that a Pneumococcal Conjugate Vaccine (PCV) be introduced in India. [3]. Initially, PCV was introduced in 5 states in India in 2017 with partial assistance from Gavi for its initial introduction [4], [5]. Based on the result of years of clinical research and the prevailing disease burden of pneumococcal disease in the country, a decision to roll out PCV in the entire country was made. However, before the vaccine could be rolled out, the COVID-19 pandemic hit the world [6], [7].

During the pandemic, several supply-side limitations, such as delayed shipments of vaccines and supplies, staff shortages, and delayed or canceled campaigns and vaccine introduction, have threatened vaccine deliveries [8]. In contrast to available data from other countries worldwide, India has successfully rolled out PCV to the entire nation during the ongoing pandemic in a record span of around 7 months [9]. Hence, it is crucial to pinpoint the critical factors that contributed to a successful scale-up of PCV in the entire nation despite the health system struggling with an ongoing pandemic. This review states the factors critical to upscaling PCV in the country during the ongoing COVID-19 pandemic.

## Methodology

This review is a retrospective study examining the critical factors enabling the successful expansion of the PCV in 31 states and UTs of the country. To generate the highest possible evidence base, a review was developed in two stages. The first stage comprised a comprehensive literature search conducted on PubMed, Cochrane database and Google Scholar. Published peer-reviewed literature, grey literature, and official guidelines and reports were scanned, selected, and reviewed. Only articles in English and on the PCV vaccine were included. Articles in other regional languages were excluded from the selection. The authors (the National EPI Manager for PCV expansion from the Ministry of Health & Family Welfare, the Donor from the Bill & Melinda Gates Foundation, and the Program Managers and Programme Officers from the lead technical agency, John Snow India) brainstormed and discussed their experiences with the PCV introduction and expansion in the second stage. The review is comprehensively dependent on the literature studied and the authors' opinions and experiences.

## Results and discussion

**Fig. 1:**
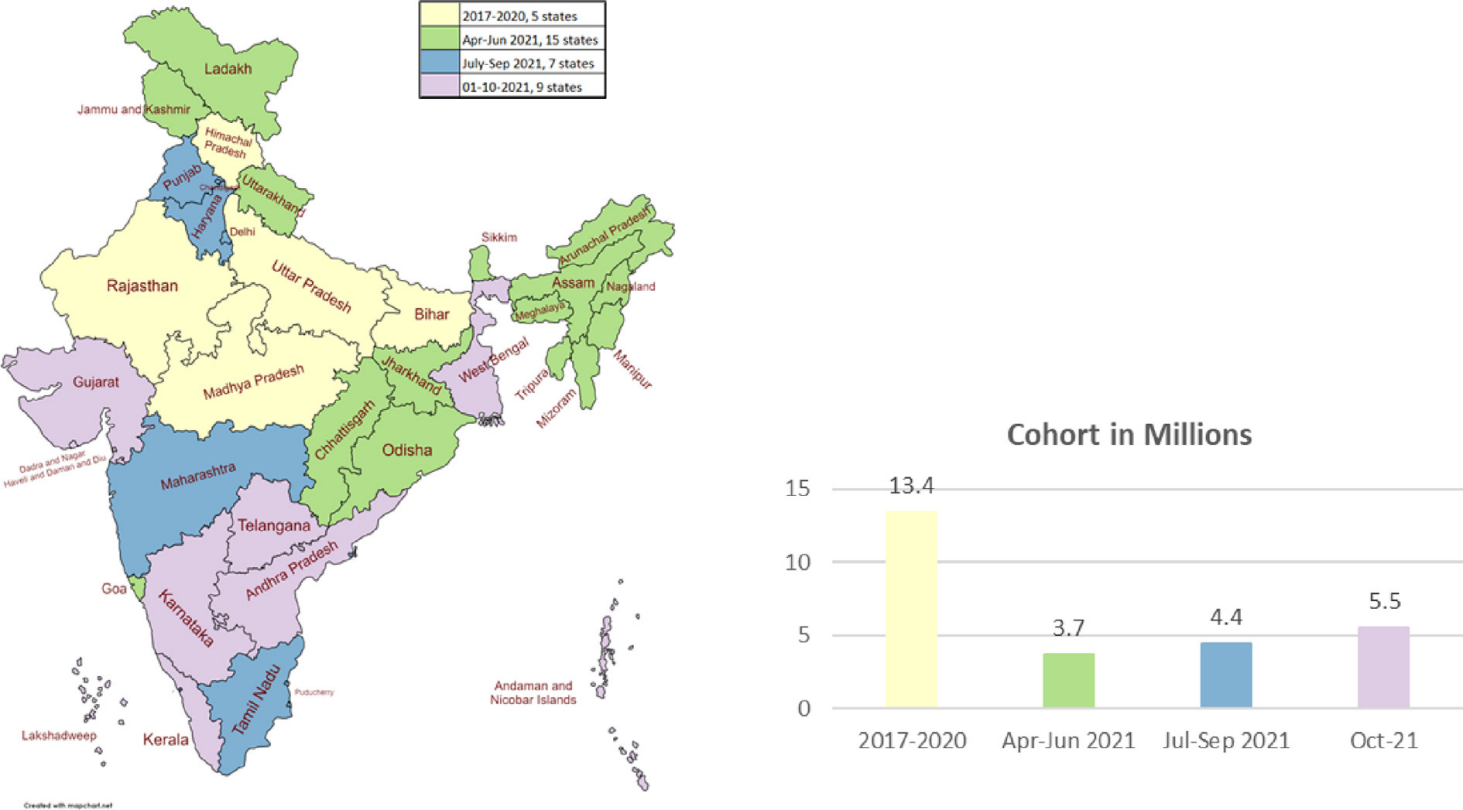
PCV-Introduction and expansion in the UIP.

PCV was the 11th vaccine to be introduced in India; however, its introduction was marked by the unprecendented challenges of an ongoing pandemic threatening health services. While the country was facing massive challenges in the form of an overburdened health system, lack of adequate human resources, and increased demand for healthcare services, PCV was expanded across 31 states and UTs of the country, covering a birth cohort of 50.4% in a duration of 7 months [10]. Fig. 1 depicts the state-wise expansion of PCV in the country.

**Fig. 2:**
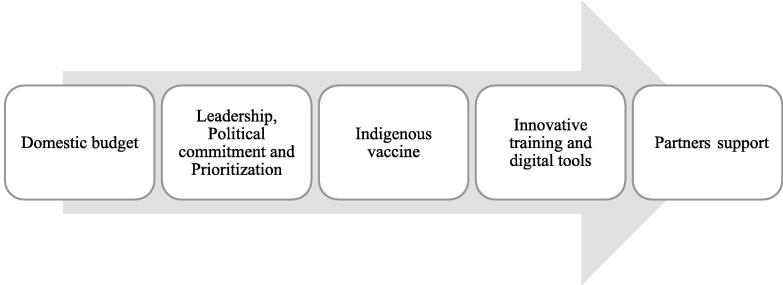
Five pillars of PCV expansion during the pandemic.

From a review of the existing literature and the opinions of the authors who coordinated and shared the global evidence and perspectives for the PCV expansion in India, five pillars of PCV introduction were derived. Fig. 2 depicts the five pillars that enabled a successful upscaling of PCV across the country.

## Domestic budget

In 2021, the Finance Minister of India, announced that PCV, which is manufactured indigenously, will be rolled out across the country and this will prevent more than 50,000 child deaths annually [11], [12].

Including PCV in India’s health budget despite the health systems being stretched in tackling the pandemic was one of the key factors which led to the rapid expansion of PCV in a very short period. The provisioning of PCV procurement through domestic budget ensured uninterrupted vaccine supply which is critical for a expeditious roll out of a new vaccine [13].

## Leadership, political commitment, and prioritization

A look at different countries reveals that flexibility in parental working patterns and enhanced awareness through the phone- and email-based reminders helped in improving vaccine uptake. In countries like Senegal, Portugal, and Sri Lanka, a well-planned media campaign emphasizing the importance of vaccination even during lockdown helped in improving vaccine delivery. Despite delays, many countries were also able to implement vaccination campaigns in 2020. All these examples point toward one common factor-a quick response from the leaders to meet the unique challenges posed by the pandemic and associated restrictions [9].

Back in 2015, following NTAGI’s recommendation, the Indian government approved the PCV within seven months, making it one of the fastest vaccines from evidence presentation to policy decision. In 2021, PCV became the first vaccine with an earmarked budget. Indian leadership has followed scientifically backed vaccination initiatives that were based on proper prioritization and principles of social justice [14].

The Indian political and health leadership demonstrated strong political commitment, stewardship, and an ability to handle difficult unprecedented situations like the COVID-19 pandemic. With the decision to expand PCV in the entire nation during the pandemic, India has set an example for the world. The decision to prioritize PCV expansion during the pandemic was compounded by the constant and persistent monitoring and interest of the Prime Minister’s Office, Cabinet, and NITI Aayog for regular progress updates. One of the biggest drivers of the successful expansion of PCV in the country was the inclusion of the PCV in the health budget of the country.

## Indigenous vaccine

Even though India introduced PCV in 5 states in 2017, there were lingering affordability issues. The PCV vaccination program is dependent on innovative financing mechanisms initiated by Gavi. With an estimated 27 million babies born each year in India and highly-priced PCVs, nationwide scale-up of the vaccine was too expensive. However, the cost element had a significant effect on the expansion of PCV in all the states.

“Pneumosil” is the first indigenously developed pneumococcal vaccine in India. It received prequalification status from the World Health Organization in 2019, was considered safe and effective, and was approved to be used in international programs run by UNICEF and Gavi [15]. In India, Pneumosil was licensed for use in 2020 [22].

It can be cited as a significant milestone in the public health care of India as it allowed for an affordable and high-quality vaccine to be offered through the universal immunization program. It is the third PCV to be certified by Gavi and is currently available at a Gavi price of US$2/dose [15]. Since the vaccine was developed and completely manufactured in the country, smooth and uninterrupted delivery of PCV could be maintained to all the states for a timely rollout of PCV, despite the movement and other associated restrictions associated with the COVID-19 lockdown. With a cost of approximately 30% less than the other PCVs available through Gavi [14], the affordability of Pneumosil allowed India to increase the pace of its plan for PCV expansion to all its remaining states.

## Innovative training and digital tools

The commitment of the political leadership in recommending PCV expansion and endorsing it in the budget 2021, the availability of the indigenous vaccine at Gavi price, had set the stage for PCV expansion. But a new challenge awaited the world, the COVID-19 pandemic. PCV rollout amidst the pandemic could have interfered with the COVID-19 vaccination campaign in the country. However, deployment took place at a remarkable speed.

It is unarguable that successful implementation of a new vaccine introduction requires an enormous effort which meant enhancing and leveraging the digital platform across the country to monitor the vaccine supply chain (i.e., eVIN, the Electronic Vaccine Intelligence Network), strengthening surveillance systems to monitor adverse immunization events, and increasing the network of cold-chain equipment. However, the large-scale procurement of new cold chain equipment countrywide in face of the COVID-19 vaccine roll-out aided the smooth expansion of PCV [16]. In addition, the health workforce involved in the PCV delivery and administration would require the training of 32,000 medical officers, 20, 000 cold-chain handlers, and more than 150, 000 vaccinators [14].

COVID-19 posed challenges due to lockdowns and restrictions on gatherings, so in-person training for PCV introduction was not possible in many places. Hence, most of the training happened online through virtual platforms. Though a cascaded model of training was adopted, the training was conducted in hybrid mode, and a training tracker was developed to monitor the status of ongoing training. Innovative and technologically adapted training packages with pre-recorded sessions from immunization experts were employed to train the workforce. With the commitment and hard work of the team that worked on COVID-19 during the day and PCV during the evenings, the entire workforce was trained within a period of 7 months. In this duration, 515 district-level training of trainers (ToTs), 3527 block-level ToTs, 31,953 medical officers, 20,000 cold chain handlers, 1.5 lakh auxiliary nurse midwives, 16, 500 accredited social health activists, and 4.5 lakh Anganwadi workers were trained [17].

A digital tool, PCV Roll Out Monitoring and Preparedness Tool (PROMPT) was conceptualized, developed, and implemented for efficient remote functioning during the pandemic. The PROMPT tool is an innovative, interactive, and user-friendly online interface for preparedness assessment which can be accessed through the PROMPT website [18]. PROMPT aimed to obtain filled assessment checklists in a pre-designed format from the states and districts through a digitalized approach, eliminating the intensive documentation and reducing the strenuous workload on human resources. The tool facilitated the easy and quick compilation, collation, and analysis of the preparedness assessment status of states and UTs. It enabled the states/UTs to identify the gaps and take appropriate corrective measures.

## Partners support

The vaccination program in India leveraged the existing strengths and identified novel approaches to mitigate disruptions and introduce PCV in 31 states and UTs in the country. India has a well-nurtured and sustainable ecosystem of partners and stakeholders working in the field of immunization. Earlier vaccination campaigns and the introduction of new vaccines have created a fertile ground for vaccine innovation, introduction, and implementation. The country’s polio-free status, successful delivery of the Indian flagship program, ‘Mission Indradhanush’ campaign, and introductions of five new lifesaving vaccines in the last decade are examples of the extensively utilized public–private partnerships in the country [19], [20], [21].

However, the scenario was different when the country upscaled the PCV from 5 states to 31 states and UTs, because of the COVID-19 pandemic and prevailing lockdowns. A 360-degree approach was adopted to highlight the need, value, safety, and time of introduction of PCV. This employed the successful utilization of both electronic and print media at all levels. Additionally, the training and IEC materials were developed in English, Hindi and 11 regional languages to facilitate the training of the health workforce across different geographies. All the health workers and immunization partners were busy with COVID-19 vaccination, but still, they provided whole-hearted support for PCV expansion. The all-round involvement of the central and ground workforce of the partners from WHO, UNICEF, UNDP, ITSU, JSI, and NCCVMRC in conceptualizing, developing, and executing a holistic approach to introducing PCV during the pandemic led to an innovative, extensive, and carefully planned program to introduce PCV.

## Conclusion

Pneumococcal Conjugate Vaccine is an Indian success story. Produced in India by an Indian company, Pneumosil is available globally to low-and middle-income countries for US$2 per dose. Despite an ongoing pandemic, where the healthcare functionaries and the government focus were on managing COVID-19, India successfully expanded PCV across all the states and union territories in a span of 7 months. Five critical factors have been key to the rollout of PCV in India, including a domestic budget for PCV, strong leadership, political commitment, and health prioritization; availability of an affordable indigenous vaccine; development and implementation of innovative training and digital tools; and all-encompassing and enthusiastic partners support.

## Declaration of Competing Interest

The authors declare that they have no known competing financial interests or personal relationships that could have appeared to influence the work reported in this paper.

## Data Availability

No data was used for the research described in the article.
